# Swallowing False Teeth

**Published:** 1883-06

**Authors:** 


### SWALLOWING FALSE TEETH.

An inquest was held at St. Bartholomew's hospital by Mr, Langham on the body of a man from Rhayader in North-Wales. who died from the effects of swallowing a small plate carrying two artificial teeth. He was brought from Wales to London, and admitted into the hospital, and an operation was performed to relieve the urgent dyspnœa, but death eventually supervened owing to an abscess caused by the impaction of the plate. There is no rose without a thorn, no advantage without a drawback. Even the blessing of false teeth to the toothless has a possible curse hidden beneath its fair exterior; the teeth may break loose from their fixings or fastenings, either when in use or while the wearer sleeps, and being either swallowed or drawn with the breath into the larynx, trachea, or bronchus, become converted from an aid to living into a cause of death. The moral of this consideration ought to be, not that the dread of being "in peril by false teeth" should frighten persons from wearing them, but that the dentists should be particularly careful to provide secure fastenings. There can be no doubt that there is a great deal of careless work in the fitting of artificial teeth. Setting aside the multitude of clumsy and inartistic teeth thrust into the mouths of the ignorant or too forbearing, there is a large proportion of cases in which safety is sacrificed to seeming comfort or appearance. It may be easy to wear a set of teeth, or to keep a single tooth in place with ordinary attention when the attachments have been reduced to a minimum, but a very little careless eating or talking may, at any moment, render such attachments of no value. We have heard dentists themselves express wonder at the fact that "sets" supplied at the special request of the wearers, with little or nothing to hold them in place, could be worn. Dentists should insist on making the teeth they apply thoroughly secure; and their clients should submit the question of fastenings, both as regards number and extent, entirely to their judgment.—*The Lancet.*

